# Could Sporadic Creutzfeldt-Jakob Disease Be Underdiagnosed in China? Experience From Four Cases

**DOI:** 10.3389/fneur.2020.00763

**Published:** 2020-07-28

**Authors:** Yi-Liu Zhang, Xiao-Mei Wu, Yang Chen, Wen-Ping Gu, Wei Lu

**Affiliations:** ^1^Department of Neurology, The Second Xiangya Hospital, Central South University, Changsha, China; ^2^Department of Neurology, Xiangya Hospital, Central South University, Changsha, China

**Keywords:** Creutzfeldt-Jakob disease, prion disease, neuroinfectious disease, PrP^SC^, rapidly progressive dementia

## Abstract

**Background:** Creutzfeldt-Jakob Disease (CJD) is a rapidly progressive neurodegenerative disease caused by the misfolded version of the cellular prion protein. Here we report four cases of sporadic CJD (sCJD) and describe the diagnostic methods available in order avoid missed or delayed recognition of CJD in China.

**Case presentation:** We report four patients diagnosed with sCJD between March 2018 and December 2019 at Xiangya Hospital and the Second Xiangya Hospital of Central South University. All patients were admitted to the hospital because of a progressive cognitive decline. Although their routine tests and biochemical indicators in the cerebrospinal fluid (CSF), as well as computed tomography (CT) imaging, did not reveal any apparent abnormalities, the presence of “cortical ribboning” was incidentally found on diffusion-weighted imaging (DWI). The patients were subsequently diagnosed with CJD based on positive testing for 14-3-3 protein in their CSF, and the presence of periodic sharp and slow wave complexes (PSWCs) on their electroencephalograms (EEG). Additionally, two of patients was confirmed pathological examination of cerebral biopsies demonstrating neuronal loss, gliosis, and spongiform changes.

**Conclusions:** CJD is a rare disease and is easily misdiagnosed by clinician in China due to a lack of recognition and awareness of CJD. Based on our experience described in this report, enhanced vigilance for CJD is required for patients with rapidly progressive dementia in China and other developing countries. DWI, EEG and detection of 14-3-3 protein in CSF should be performed in order to achieve a timely diagnosis of CJD.

## Introduction

Creutzfeldt-Jakob Disease (CJD) is a rapidly progressive neurodegenerative disease caused by the misfolded version of the cellular prion protein referred to as PrP^SC^, resulting in neuronal loss, spongiosis, and astrogliosis. It is considered a rare disease compared to other neurodegenerative disorders. Sporadic CJD (sCJD) is commonest form of CJD. In addition to rapidly progressive dementia, other symptoms can also frequently occur in CJD patients, such as myoclonus, cortical blindness, ataxia, and akinetic mutism, which are non-specific for CJD and thus often cause diagnostic difficulties for clinicians. Generally, routine tests such as cerebral computed tomography (CT), T1- and T2-weighted magnetic resonance imaging (MRI) scans, and standard cerebrospinal fluid (CSF) testing cannot provide diagnostic information for CJD. Although spongiform changes on brain biopsy is considered the only gold standard for the definitively diagnosing CJD, brain biopsy has low practicability, which is likely to be a reason why CJD is difficult to recognize and easy to misdiagnose in China and in other countries with limited access to more advanced diagnostic tools or with little knowledge of CJD. However, with the development of diagnostic techniques such as fluid-attenuated inversion recovery (FLAIR) MRI, diffusion-weighted imaging (DWI), specific analyses of CSF biomarkers, electroencephalography (EEG), and other new methods, an early clinical diagnosis of sCJD can be made without the need for a brain biopsy.

In conclusion, CJD is classified as a rare disease possibly and partly because of the poor understanding about CJD among clinicians, limited diagnostic methods, and the high rate of misdiagnosis in developing countries.

## Cases Presentations

We retrospectively reviewed the medical records of patients diagnosed with CJD between March 2018 and December 2019 at Xiangya Hospital and the Second Xiangya Hospital of Central South University. Only patients aged 18–90 years with CJD who met the proposed diagnostic criteria were included (1). The information collected and analyzed from patients included: sex, age at onset of CJD, age of death, disease duration, symptoms at onset, and examination findings.

The clinical features of the patients are summarized in Table 1. Ages at onset of CJD ranged from 60 to 66 years (mean age was 61.75 years), and the group included two women and two men. They did not have any history of contacting a source of infection or related CJD family history. All patients experienced characteristics of a progressive dementia within <2 years; for example, patients 1, 3, and 4 did not recognize their family members and could not recall their home address. Moreover, all patients had myoclonus, ataxia, and extrapyramidal or pyramidal signs during disease progression, and either visual disturbances or akinetic mutism developed in the other two patients. All patients were admitted to hospital with obvious symptoms of dementia.

**Table 1:** Clinical features and results of assistant examination in patients.

	**Patient 1**	**Patient 2**	**Patient 3**	**Patient 4**
Age at onset of CJD/of death	61/61	60/61	66/67	60/60
Sex	Male	Female	Male	Female
Occupation	Farmer	Farmer	Farmer	Farmer
Time interval between symptom onset and death (months)	6	4	12	8
Symptoms at onset	Dizziness and nausea	Dizziness and memory impairment	Severe headache and dizziness	Memory impairment and personality change
Symptoms during evolution	Walking unstable, involuntary movement of the limbs, rapidly progressive dementia	Psychological and behavioral abnormalities, involuntary movement of the limbs	Memory impairment, vision loss of left eye from	Difficulty in walking, occasional myoclonic spasm (lasted 1 or 2 s)
CT	NP	–	NP	–
Cerebral lesion where had abnormal high signal in DWI	Bilateral frontal parietal occipital lobe, left temporal lobe, bilateral caudate nucleus and putamen	White matter of anterior, posterior horn of bilateral ventricle, bilateral temporal lobe, and occipital lobe	White matter of anterior, posterior horn of bilateral lateral ventrical and centrum semiovale, bilateral temporal lobe	Bilateral occipital, temporal cortex, and subcortical
PSWCs on EEG	+	–	+	±
Result of 14-3-3 in CSF	+	+	+	+
Pathologic examination of brain	Spongiform change	NP	NP	Spongiform change
PrP^sc^ protein in brain	+	NP	NP	+
PRNP	No pathogenic mutation	No pathogenic mutation	No pathogenic mutation	No pathogenic mutation

*NP, not performed; CSF, Cerebrospinal fluid; CT, Computed tomography; DWI, Diffusion-weighted imaging; FLAIR, Fluid-attenuated inversion recovery; PSWCs, periodic sharp and slow wave complexes; EEG, Electroencephalograms*.

**Figure 1:**
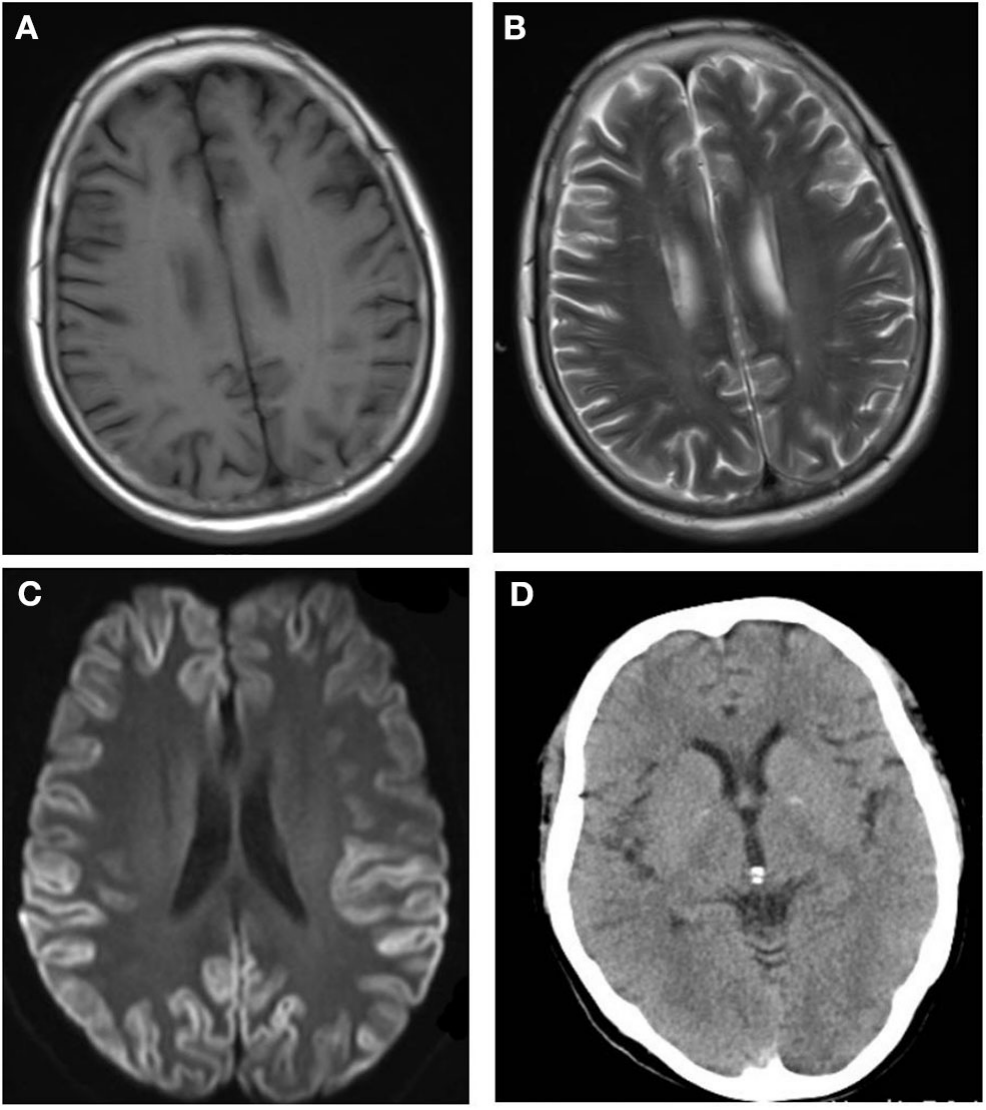
No abnormalities are detectable on the T1-weighted **(A)** and T2-weighted **(B)** magnetic resonance imaging (MRI) sequences. The diffusion-weighted imaging (DWI; **C**) shows the typical features of “cortical ribboning” in the cortex and subcortex of bilateral frontal, temporal, and occipital lobes on DWI **(C)**. On the apparent diffusion coefficient map (ABC; **D**), there is low signal intensity in the cortex and subcortex of the bilateral frontal, temporal, and occipital lobes.

**Figure 2:**
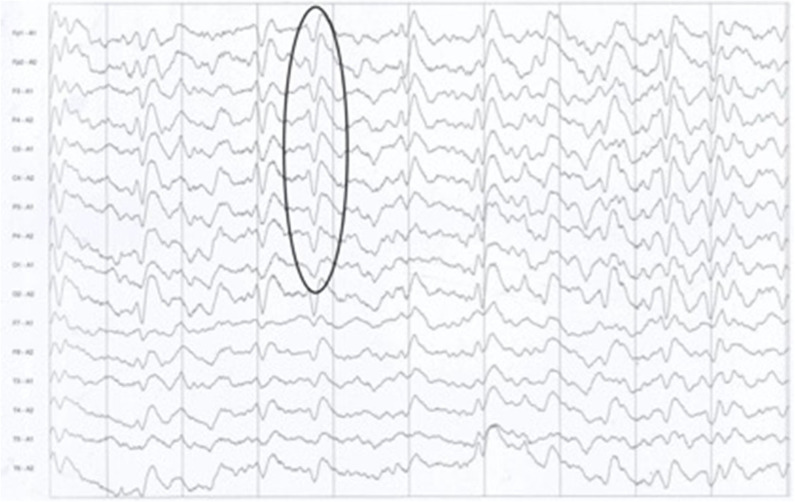
The typical presence of periodic sharp wave complexes (PSWCs) on the electrocephalogram of patient 1.

After admission, cerebral CT as well as T1- and T2-weighted cerebral MRI were performed in all patients, and no apparent abnormalities were identified. Lumbar puncture was also performed, and there were no positive results in the routine laboratory tests, biochemical indicators, complete viral testing, histologic staining, oligoclonal bands (OB) testing, or autoimmune encephalitis antibodies in CSF. However, abnormal high signal in two or more cerebral cortex regions, referred to as “cortical ribboning” (Figure 1), and/or the basal ganglia, was incidentally found in the DWI of all four patients, which alerted physicians to suspect a diagnosis of CJD. These patients were all subsequently recommended to undergo the following tests: DWI, EEG, and laboratory CSF analysis; two of them also underwent a brain biopsy. Testing performed by China DCD (Center for Disease Control and Prevention) for CSF 14-3-3 protein were positive in all patients. However, tests for other CSF biomarkers such as total tau protein, S100B, neuronal specific enolase and amyloid-β (Aβ)1-42 were not performed because these assays were not widely used in Chinese hospitals at the time. Patients 1 and 3 had characteristic period sharp wave complexes (PSWCs; Figure 2) recorded by EEG. And atypical little sharp waves, instead of PSWCs, were identified in the EEG trace of patient 4. Additionally, upon consideration of the risk of brain surgery, only two patients (#1 and #4) underwent a brain biopsy, which in both cased demonstrated prominent spongiform changes and neuronal loss in the gray matter of the brain. PrP^Sc^ protein was also detected in the living brain tissue by western blotting. Moreover, prion protein (PRNP) genetic testing in the four patients did not reveal any pathogenic mutation. In light of the typical clinical features, the presence of “cortical ribboning” on DWI, 14-3-3 protein in CSF, PSWCs on the EEG, and the results of brain biopsy, all four patients were diagnosed with sCJD; two (#1, #4) were confirmed cases and the other two (#2, #3) were probable cases. There were no specific treatments available for CJD. The average time interval between the onset of CJD symptoms and death ranged from 4 to 12 months (average: 7.5 months).

## Discussion

Prion diseases are rare with a poor prognosis, with a reported incidence of 1.5–2 cases per million people per year (2). The clinical manifestations of sCJD at onset are variable and non-specific, but rapidly progressive dementia is a necessity according to the above diagnostic criteria (1), and indeed it was evident in all four patients described in this report. In addition to persistent finding of cognitive decline, visual disturbances, ataxia, extrapyramidal or pyramidal signs, myoclonus, and akinetic mutism could appear as major symptoms, consistent with the clinical features of our four patients. As a result, the CJD is often easily missed or incorrectly diagnosed. The two most common differential diagnoses of CJD are Alzheimer's disease and vascular dementia. Other relatively treatable dementia conditions, including immune-mediated disorders (such as autoimmune encephalitis), neoplasia (most often lymphoma), infections, and metabolic disorders (3, 4), are less likely to be misdiagnosed as CJD because they are more common. Therefore, when meeting patients with symptoms of dementia, doctors should suggest that these patients complete some examinations in order to further distinguish CJD from these treatable dementia conditions.

According to the World Health Organization (5), brain biopsy is the only tool for obtaining a definitive diagnosis of CJD, but it is hardly accepted by patients' families and it is less used clinically due to its invasiveness. It has been reported that there were 1,464 cases of CJD and 1,302 cases of sCJD between 2006 and 2017 in China (6). Among these cases of sCJD, only 0.31% (4/1302) were definitive diagnostic cases (6). Nonetheless, scientific and technical advances have facilitated great progress in the development of non-invasive methods for diagnosing sCJD, which may be one of the reasons why the reported incidence of CJD has increased each year in China (6). Compared with cerebral CT, DWI and FLAIR MRI are considered sensitive imaging tools for making the clinical diagnosis of CJD worldwide (7). The typical characteristics of sCJD in DWI or FLAIR MRI are: restricted diffusion either in the basal ganglia alone (caudate and/or putamen) or in more than one cortical region, also known as “cortical ribboning,” and the absence of gadolinium enhancement (8). Typical “cortical ribboning” features on DWI in the four patients of our study indicated a suspected diagnosis of CJD. Therefore, for patients with a rapidly progressive dementia, brain DWI and FLAIR MRI should be used to differentiate sCJD from other causes of rapidly progressive dementia, which could contribute to an early diagnosis.

A previous study indicated that development of PSWCs in CJD is associated with the disease course and is consistent with the severity of brain injury (9). Furthermore, PSWCs are usually seen during the advanced stages of CJD and may disappear during later stages with the deterioration of brain function. On average, there is a 3.7-month delay from disease onset to the manifestation of PSWCs findings on EEG (9). For example, patient 2 survived 4 months because of a serious and rapidly progressing condition. Her EEG had an absence of PSWCs, which may have resulted from her EEG being performed in the late phase of the disease. Although PSWCs are important, other diseases such as Alzheimer's disease, dementia with Lewy bodies, and vascular dementias also need to be considered in the differential diagnosis, when PSWCs are recorded in patients with rapidly progressive dementia (9).

Generally, routine and biochemical CSF tests in sCJD patients are usually normal, which might explain why physicians often ignore the potential diagnosis of CJD. Several CSF biomarkers play a significant role in the diagnosis of sCJD, including 14-3-3 protein, tau protein, phosphorylated tau, S100 protein, neuronal specific enolase, and amyloid-β (Aβ)1-42, either alone or in combination (10–14). Among these biomarkers, the detection of the 14-3-3 protein is the most commonly used method (7). In this study, after typical changes were observed on the brain DWI, the examination of 14-3-3 proteins in CSF was completed immediately and the results were positive. Furthermore, no mutations were found in the PRNP gene sequencing analysis performed in the four patients. However, 14-3-3 protein is a non-specific biomarker of neurodegeneration that appears after neuronal destruction, which can occur during infections, inflammatory events, stroke, epileptic seizures, toxic-metabolic conditions, and other types of dementias (15, 16). Some studies show that the detection 14-3-3 protein in CSF combined with the quantification of tau protein, a microtubule-binding polypeptide highly expressed in neurons and their axonal projections, is particularly useful in the differential diagnosis of rapidly progressive dementia (17–19). In addition, Zanusso et al. proposed a diagnostic flow-chart combined with the determination of the presence of 14-3-3, quantification of total tau, ratio of Aβ1-42/phosphor-tau181 and ratio of phosphotau181/total tau, which might help to differentiate sCJD from other diseases (20). Unfortunately, these CSF assays were not performed in this study because they were not widely used in Chinese hospitals at the time, which further reinforces the difficulties in diagnosing CJD and a lack of awareness about CJD among physicians in China and other developing countries. Regardless, a positive test result for 14-3-3 protein in the CSF is still highly suggestive of a CJD diagnosis when accompanied by the presence of PSWCs on the EEG and distinctive DWI/FLAIR MRI changes.

Recently, some less invasive examination techniques for CJD described in some epidemiological diagnostic studies, one of which is real-time quaking-induced conversion (RT-QuIC) that can detect minute amounts of misfolded prions in the CSF, olfactory mucosa (OM), and skin samples, by amplifying PrP^SC^ using recombinant PrP as a substrate (21). This is increasingly becoming a potential alternative diagnostic tools, as it is a less invasive method for obtaining a definitive diagnosis, with recent investigations showing virtually 100% diagnostic sensitivity and specificity in a proposed diagnostic algorithm of a combining the CSF and OM RT-QulC for patients with sCJD (22). Although CSF and OM RT-QuIC testing is available in most western countries, as well as Japan and Australia, and it appears feasible in clinical practice (21), it has not yet been widely introduced in Chinese hospitals. This could be contributing to an increased the difficulty in diagnosing CJD in China and other developing countries. Moreover, recent animal research has indicated that skin PrP^SC^ via RT-QulC and serial protein misfolding cyclic amplification (sPMCA) may also be a useful method in the future for the preclinical diagnosis of prion diseases as well as monitoring disease progression following infection and treatment (23).

## Conclusion

CJD is a rare disease, but the limited availability of more advance diagnostic methods combined with a lack of awareness about CJD in China and other developing countries may be leading to a missed diagnoses, misdiagnoses and low diagnostic rate. New, less invasive and more accessible diagnostic techniques as an alternative to brain biopsy, including EEG, DWI, RT-QuIC, 14-3-3 protein, and other biomarkers, could improve the accuracy of diagnosing prion diseases. We diagnosed four cases within a <2-year period, which alerts us to CJD being a public health concern that requires greater attention. In China, awareness should be generated for patients with rapidly progressive dementia in clinical practice, and patients should be examined by methods such as DWI, testing for 14-3-3 proteins in the CSF, RT-QuIC, and so on, in order to ensure a correct diagnosis.

## Data Availability Statement

All datasets generated for this study are included in the article/supplementary material.

## Ethics Statement

Written informed consent was obtained from the individuals for the publication of any potentially identifiable images or data included in this article.

## Author Contributions

Y-LZ wrote the manuscript and prepared the table/figures. WL and W-PG co-conceived this study and designed the experiments. Y-LZ, YC, and X-MW collected and analyzed clinical data. All authors read and approved the final manuscript and agreed to submit it for publication.

## Conflict of Interest

The authors declare that the research was conducted in the absence of any commercial or financial relationships that could be construed as a potential conflict of interest.

## Abbreviations

CSF: Cerebrospinal fluid
CT: Computed tomography
DWI: Diffusion-weighted imaging
EEG: Electroencephalograms
FLAIR: Fluid-attenuated inversion recovery
OB: Oligoclonal bands
PSWCs: periodic sharp and slow wave complexes
RT-QuIC: Real-time quaking-induced conversion sCJD, sporadic Creutzfeldt-Jakob disease.
